# Streamline-based three-dimensional peak-velocity tracing of transvalvular flow using four-dimensional flow cardiac magnetic resonance imaging for left ventricular diastolic assessment in aortic regurgitation: a case report

**DOI:** 10.1186/s13256-022-03422-7

**Published:** 2022-05-16

**Authors:** Paul Njoku, James Wardley, Pankaj Garg

**Affiliations:** 1 Cardiology, Norfolk and Norwich University Hospital NHS Trust, Norwich, UK; 2 Norwich Medical School, University of East Anglia, Norwich, NR4 7TJ UK

**Keywords:** Aortic valve insufficiency, Hemodynamics, Echocardiography, Magnetic resonance imaging, Aortic regurgitation, Diastolic function, 4D flow CMR, Mitral inflow

## Abstract

**Background:**

Doppler transthoracic echocardiography is routinely performed to measure peak mitral inflow velocities in the assessment of left ventricular diastolic function. The limitations of echocardiography are well documented, but its accuracy in the measurement of transmitral peak velocity in the presence of aortic valve regurgitation has not yet been compared with four-dimensional flow cardiac magnetic resonance imaging. Four-dimensional flow cardiac magnetic resonance imaging offers time-resolved cross-sectional velocity information that can be used to investigate mitral inflow peak velocity. We present a case report demonstrating the potential superior capabilities of four-dimensional flow cardiac magnetic resonance imaging in accurately detecting mitral inflow velocities over Doppler echocardiography in patients with aortic regurgitation.

**Case presentation:**

A 67-year-old Caucasian female presented to our outpatient cardiology clinic with exertional dyspnea. Doppler transthoracic echocardiography identified moderate to severe aortic regurgitation. Mapping of mitral inflow peak velocities proved challenging with Doppler echocardiography. Additionally, four-dimensional flow cardiac magnetic resonance imaging with automated three-dimensional flow streamlines was performed, which allowed for more accurate detection of mitral inflow peak velocities.

**Conclusions:**

Doppler echocardiography has a limited role in mitral inflow assessment where aortic regurgitation is present. In such cases, four-dimensional flow cardiac magnetic resonance imaging is an alternative imaging technique that may circumvent this issue and allow mitral inflow assessment.

**Supplementary Information:**

The online version contains supplementary material available at https://doi.org/10.1186/s13256-022-03422-7.

## Background

Doppler echocardiography is a widely accessible cardiac imaging tool used routinely in the assessment of mitral inflow peak velocity [1]. Peak E-wave (peak velocity of transmitral blood flow in early LV diastole) and A-wave (peak mitral inflow velocity in late diastole due to atrial contraction) velocities can be measured using pulsed-wave Doppler echocardiography with good feasibility and reproducibility [1]. These velocity parameters are frequently used as surrogate markers of left ventricular (LV) diastolic function. E/A ratio can then be calculated to define and classify diastolic function in patients with heart failure symptoms not explained by impaired systolic function or reduced ejection fraction [1]. The pathophysiology of heart failure with preserved ejection fraction (HFpEF) is related to impaired LV compliance and relaxation causing diastolic dysfunction. HFpEF is commonly overlooked or misdiagnosed [2], therefore it is imperative that reliable, noninvasive imaging modalities be investigated to accurately assess LV diastolic function, particularly in certain subgroups of patients. The use of pulsed-wave echocardiography is hampered by limitations associated with poor acoustic windows and operator dependence [3]. Four-dimensional (4D) flow cardiac magnetic resonance imaging (CMR) offers time-resolved cross-sectional velocity information [4] that can be used to investigate mitral inflow peak velocity where echocardiography is not suitable. Recent studies have reported good agreement between 4D flow CMR and Doppler echocardiography measurements of mitral E-wave and A-wave velocities [5, 6]. It has been documented that, in cases of severe aortic regurgitation (AR), the regurgitant jet can interfere with mitral inflow velocity readings [1], making echocardiographic detection challenging. To the best of our knowledge, no previous literature has reported on the utility of 4D flow CMR over Doppler echocardiography in deriving mitral inflow velocity, in cases of aortic valve regurgitation. Here we present a case where 4D flow CMR was used to better determine mitral inflow peak velocities in a patient where aortic valve regurgitation complicated Doppler-derived measurements.

## Case report

**Fig. 1:**
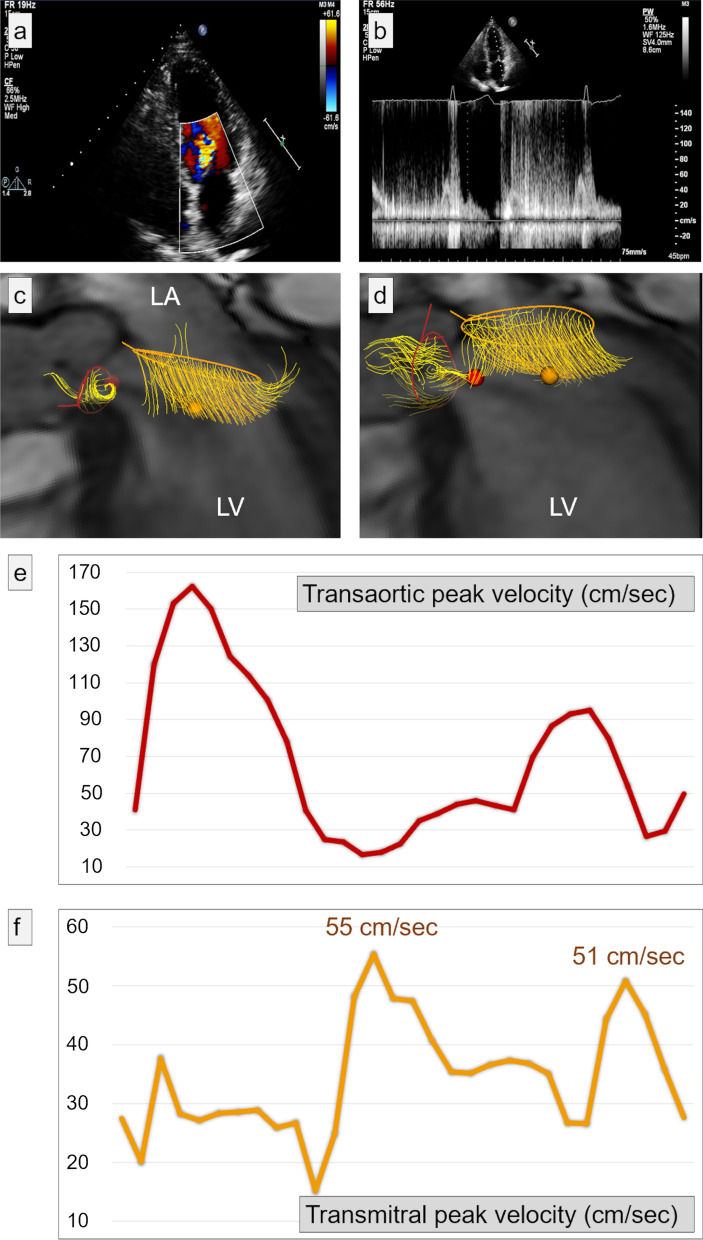
Mitral inflow assessment of peak early (E-wave) and late (A-wave) inflow velocities. **a**, **b |** Transthoracic echocardiography: turbulent aortic regurgitation demonstrated in the apical 2 chamber (**a**). Pulsed-wave Doppler echocardiography at the tip of the mitral valve leaflet results in very turbulent early filling which prohibited peak mitral inflow E-wave velocity assessment. **c**, **d** On 4D flow CMR assessment by peak velocity tracing within the three-dimensional space of the streamlines, the peak velocity during early filling (**c**) and late filling (**d**) were recorded by limiting the streamline assessment to length of the mitral valve leaflets. The peak velocity is depicted as the orange ball for mitral inflow and as red ball for aortic regurgitation in the three-chamber view. **e**, **f** Both transaortic and transmitral peak velocity traces are presented. Compared with pulse-wave echocardiography, a clearer depiction of peak velocity is seen for both E-wave and A-wave

In 2021, a 67-year-old Caucasian woman presented to our outpatient cardiology clinic with worsening exertional breathlessness with no physical stigmata of heart failure. Transthoracic echocardiography (TTE) demonstrated an aortic regurgitation (AR) with a pressure half-time of 597 ms, a vena contracta of 0.45, but visual assessment was graded as moderate to severe AR (Fig. 1a, b, Additional file 1: Video S1). A cardiovascular magnetic resonance (CMR) with four-dimensional flow (4D flow) was done to better quantify the AR and the left ventricular (LV) volumes. CMR was performed on a 3-T Discovery 750w GE system (GE Healthcare, Milwaukee, WI, USA) equipped with an eight-channel cardiac coil. CMR cine images in two-, three-, and four-chamber views were obtained during end-expiratory breath-hold with a balanced steady-state free precession (bSSFP), single-slice breath-hold sequence. Images encompassed the entire heart, aortic valve, and ascending aorta using the following scan parameters: HyperKat acceleration with a factor of 2, field of view 340 mm × 340 mm, acquired voxel size 3 × 3 × 3 mm^3^, reconstructed voxel size 1.5 × 1.5 × 1.5 mm^3^, echo time (TE) 3.5 ms, repetition time (TR) 10 ms, flip angle 10°, and 30 cardiac phases. Four-dimensional flow analysis of peak mitral inflow velocity was performed using CAAS MR software (prototype version 5.2; Pie Medical Imaging, Maastricht, the Netherlands).

The LV was not dilated (indexed LV end-diastolic volume 84 ml/m^2^ and end-systolic volume 26 ml/m^2^) and had preserved function (ejection fraction 69%). CMR elucidated the AR severity to be mild to moderate (AR fraction 22% with no holo-diastolic reversal in descending aorta). We used novel three-dimensional peak velocity tracing of the transvalvular flow streamlines to investigate whether we could map out the mitral inflow peak diastolic early (E-wave) and late (A-wave) velocities, which were challenging to assess by pulsed-wave Doppler echocardiography (Fig. 1c, d, Additional file 2: Video S2). The flow streamlines can track blood flow from the mitral annulus, and hence there is less chance of picking up the peak velocity of aortic regurgitation flow. Even though diastolic mitral flow is likely to be blunted by the AR, this method allows to at least assess peak velocity through the mitral valve leaflets (Fig. 1e, f). This case highlights that pulsed-wave Doppler echocardiography has a limited role in the assessment of mitral inflow in the presence of AR, limiting its use in LV diastolic and mitral stenosis assessment when AR is present. Secondly, we show for the first time that 4D flow CMR plays a complementary role in teasing out the peak mitral inflow velocities in patients with AR.

## Discussion

Accurate quantification of LV diastolic function is paramount in the diagnosis, classification, and treatment of heart failure, particularly in patients with HFpEF. Pulsed-wave Doppler echocardiography is a relatively quick, low-cost, and widely accessible tool often used routinely in clinical practice to evaluate mitral inflow peak velocities and LV filling pressures [7, 8]. The quality of images obtained from echocardiography is largely dependent on operator expertise, and the consistency of findings is hampered by operator variability [3]. Other limitations of echocardiography include Doppler misalignment, limited acoustic windows, limited accuracy and reproducibility, and inferior image resolution relative to CMR [9].

This case report demonstrates the challenges associated with echocardiography in the assessment of mitral inflow peak velocities where AR is present. AR describes a valvular pathology where the aortic valve fails to close adequately [10]. During LV diastolic filling, both mitral inflow and regurgitant blood from the incompetent aortic valve contribute to the LV volume. A recent study investigated the flow dynamics in the LV of patients AR [11]. It demonstrated that as the severity of regurgitation increased, the “diastolic vortex“ generated from the aortic regurgitant jet interacted with the “vortex“ originating from true mitral inflow, competing for space in the LV cavity. As the regurgitation worsens, the mitral inflow “vortex” becomes confined to the LV wall while the regurgitant jet dominates the center of the LV chamber. It is therefore possible that the Doppler probe incorrectly detected mitral inflow, which mostly comprised blood flow from the aortic regurgitant jet. This could explain the anomalous velocity tracing depicted in this case report, which is uncharacteristic of typical mitral inflow.

Other studies have postulated a number of different mechanisms as to why this occurs in Doppler echocardiography. Enlargement of the mitral valve leaflet and left ventricle in response to chronic aortic regurgitation [12] could alter the hemodynamics of transmitral blood flow, although the exact mechanism for this remains unknown. A functional mitral stenosis may arise as a result of the aortic regurgitant jet forcing closed the mitral valve leaflets prematurely [12] or the regurgitant jet causing a “kinematic obstruction” between the mitral valve and LV apex [13], hampering transmitral inflow and the ultrasonographic detection of peak velocities across the mitral valve. These changes in the flow physics of the LV, mitral valve morphology, and function as a result of interference from the regurgitant jet could explain the challenges in accurately distinguishing peak E-wave and A-wave velocities using pulsed-wave Doppler echocardiography.

Phase-contrast 4D flow CMR is an imaging technique used to assess and visualize multidirectional blood flow in three dimensions (3D) resolved in time [4, 14, 15]. Four-dimensional flow CMR offers a promising alternative to echocardiography in LV diastolic function assessment, which we have discovered circumvents its limitations in AR. Its routine use has increased over the past few decades in the assessment of cardiac morphology, contractility, and myocardial perfusion. Studies have shown reproducibility and accuracy equal or superior to echocardiography in the assessment of mitral inflow velocities [5, 16, 17]. Four-dimensional flow CMR is less prone to operator-dependent variability and can provide greater imaging detail than standard echocardiography, but its widespread clinical use is hampered by its long scan and postprocessing times [18]. A study by Dyvorne *et al.* [19] demonstrated an accelerated 4D flow MRI technique using a combination of spiral sampling and dynamic compressed sensing to significantly reduce scan times for the quantification of blood flow in abdominal vasculature. Further investigation into how this method can translate into intracardiac blood flow quantification would be beneficial to circumvent the long scan times that are inherent to 4D flow CMR. Additionally, future study into possible technological advancements that allow further automation of 4D flow CMR analysis could reduce postprocessing times and user interference.

Here, 4D flow CMR with valve tracking and automated 3D streamline capabilities allowed better feasibility in the detection of peak mitral inflow velocities by restricting the streamline assessment to the length of the mitral valve leaflets. This capability to manually isolate mitral inflow streamlines during 4D flow mapping is unique to 4D flow CMR [20]. This allowed clearer depiction of E-wave and A-wave transvalvular peak velocities (panel F) by limiting the interference of aortic regurgitant velocities on the acquired mitral velocity measurements. This case illustrates the feasibility of 4D flow CMR in identifying mitral inflow peak velocities and LV diastolic function in patients with aortic regurgitation.

## Conclusion

This case report demonstrates for the first time the use of 4D flow CMR to circumvent the challenges associated with aortic valve regurgitation and echocardiography-derived mitral inflow peak velocities. Doppler echocardiography remains an invaluable noninvasive imaging tool in the assessment of LV diastolic function. Where echocardiography falls short, 4D flow CMR offers an attractive alternative, particularly in cases where aortic valve disease complicates echocardiographic interpretation of transmitral velocity parameters. Additional case studies would be beneficial to support the adoption of routine clinical use of 4D flow CMR in patients with aortic regurgitation undergoing LV diastolic evaluation.

## Supplementary Information

**Additional file 1: Video S1. **Video demonstrating turbulent aortic regurgitation using transthoracic echocardiography in the apical 2-chamber view. Presence of aortic regurgitation causing turbulent early-filling of mitral inflow.

**Additional file 2: Video S2.** Video demonstrating a 3-chamber view of the heart using 4D flow CMR. Peak velocity was assessed within the three-dimensional space of the streamlines, during early and late filling. The peak velocity is depicted as the orange ball for mitral inflow and as red ball for aortic regurgitation.

## Data Availability

The dataset analyzed during this case report is available from the corresponding author on reasonable request.
